# A Review on the Construction of Carbon-Based Metal Compound Composite Cathode Materials for Room Temperature Sodium-Sulfur Batteries

**DOI:** 10.3389/fchem.2022.928429

**Published:** 2022-06-09

**Authors:** Xueyu Wang, Daying Guo, Lin Yang, Minghuan Jin, Xi’an Chen, Shun Wang

**Affiliations:** Key Laboratory of Carbon Materials of Zhejiang Province, College of Chemistry and Materials Engineering, Wenzhou University, Wenzhou, China

**Keywords:** temperature sodium-sulfur battery, polysulfide shuttle, adsorption, electrocatalysis, sulfur cathodes

## Abstract

Room temperature sodium-sulfur batteries are one of the most attractive energy storage systems due to their low cost, environmental friendliness, and ultra-high energy density. However, due to the inherent slow redox kinetics and the shuttle of polysulfides, the road of room temperature sodium-sulfur batteries to practical application is still full of difficulties. As a sulfur cathode, which is directly related to battery performance, a lot of research efforts have been devoted to it and many strategies have been proposed to solve the shuttle effect problem of sulfur cathodes. This paper analyzes the existing problems and solutions of sodium-sulfur batteries, mainly discusses and summarizes the research progress of constructing carbon-based cathode materials for sodium-sulfur batteries, and expounds the current research popular from two main directions. That is to construct advanced cathode materials based on two mechanisms of adsorption and electrocatalysis. Finally, the research direction of advanced sodium-sulfur batteries is prospected.

## 1 Introduction

The advantages of the secondary battery electrochemical energy storage system, including its large capacity, good performance, long cycle, low cost, environmental protection and other characteristics, it has received extensive attention from many researchers in recent years (Sungjemmenla, et al., 2021). The widespread use of these batteries in portable electronics, electric vehicles, and energy storage stations greatly facilitates our daily lives. These energy storage devices include capacitors, fuel cells, lithium-ion batteries, and sodium-ion batteries (Huang, et al., 2021a; Li, et al., 2021a; Li, et al., 2021b). However, with the increasing global requirements for large-scale energy storage systems, some current energy storage systems with mature technologies cannot meet the needs of society. It is urgent for researchers to develop a new type of large-scale energy storage system powerful energy storage system (Ye, et al., 2020; Xiao, et al., 2021a; Xue, et al., 2022).

At present, commercial lithium-ion batteries based on conventional intercalation cathode materials and graphite anode are almost close to their theoretical energy density reaching a certain threshold, and it is of no significance to continue research (Zhong, et al., 2018; Soni, et al., 2021). Among other battery types, S-based batteries show very prominent advantages. For example, lithium-sulfur (Li-S) batteries, sodium-sulfur (Na-S) batteries and other metal-sulfur-based batteries, their ultra-high theoretical capacity and energy density are profound significance for the development and research of large-scale energy storage systems (Li, et al., 2019; Liu, et al., 2022a). As far as advanced lithium-sulfur batteries are concerned, they have a high theoretical energy density of 2,600 Wh kg^−1^ (Yang, et al., 2021a), which is expected to replace the bottleneck of lithium-ion batteries in terms of energy density. However, due to the limitation of lithium metal resources and the unfriendly price, the large-scale and large-scale promotion of lithium-sulfur batteries is limited. Fortunately, sodium metal resources are relatively abundant, and are almost inexhaustible resources. The corresponding price advantage and environmental protection characteristics make sodium-ion batteries a logical substitute for lithium-ion batteries (Wang, et al., 2020). At the same time, the physical and chemical properties of sodium element are very similar to lithium, which provides a great reference and guidance for us to study the application of sodium element in batteries.

Recently, Na-S batteries have received increasing attention in recent years due to their low cost and high energy density (2,600 Wh kg^−1^) similar to Li-S batteries (Ghosh, et al., 2020). It is considered to be one of the next-generation potential high-energy energy storage systems. However, the traditional high-temperature Na-S batteries work at a temperature of 300°C, which increases more energy losses, additional energy losses, and certain dangers (Gross and Manthiram, 2019). These problems limit its practical application (Li, et al., 2021c). In contrast, room temperature sodium-sulfur (RT Na-S) batteries have higher energy density (1,276 Wh kg^−1^), lower risk and lower cost, so the research on RT Na-S batteries using organic solvents as electrolytes has gone a long way into people’s future (Wang, et al., 2021a). Currently, the liquid electrolytes used in RT Na-S batteries are mainly carbonates and ether-based electrolytes (Luo, et al., 2016). Different from high-temperature and medium-temperature sodium-sulfur batteries, the active sulfur of RT Na-S batteries can theoretically be converted into high-capacity products (Na_2_S) (Li, et al., 2020)through multi-step reduction reactions during discharge. The reaction process is shown in Eqs. 1–5. (Yu and Manthiram, 2014; Liu, et al., 2019; Tabuyo-Martinez, et al., 2021).

$$S_{8}+2Na^{+}+2e^{-}-Na_{2}S_{8}\left(2.2\;V\right)$$ (1)

$$Na_{2}S_{8}+2Na^{+}+2e^{-}-2Na_{2}S_{4}\left(2.2∼1.65\;V\right)$$ (2)

$$3Na_{2}S_{4}+2Na^{+}+2e^{-}-4Na_{2}S_{3}\left(1.65\;V\right)$$ (3)

$$Na_{2}S_{4}+2Na^{+}+2e^{-}-2Na_{2}S_{2}\left(1.65\;V\right)$$ (4)

$$Na_{2}S_{2}+2Na^{+}+2e^{-}-2Na_{2}S\left(1.65∼1.2\;V\right)$$ (5)

Among them, Na_2_S_8_ and Na_2_S_4_ are both soluble in liquid and water in organic electrolytes, while S_8_, Na_2_S_3_, Na_2_S_2_ and Na_2_S are insoluble in organic electrolytes (Qiang, et al., 2017; Liu, et al., 2021). Therefore, the sulfur electrode undergoes a “solid-liquid-solid” phase transition process during discharge (Liu, et al., 2022b). First, sodium ions are detached from the negative electrode and shuttle to the sulfur electrode to generate long-chain polysulfides (Na_2_S_8_ and Na_2_S_4_) (Zhong, et al., 2021). Then, the long-chain polysulfides are further reduced to short-chain polysulfides, of which Na_2_S is the most desirable final discharge product (Zhou, et al., 2020). Since the reversible capacity of the battery depends on the redox reaction of polysulfides (NaPSs) (Zheng, et al., 2021), the higher the amount of Na_2_S generated, the greater the reversible capacity of the battery. The theoretical capacity of 1,675 mAh g^−1^ can only be achieved when all long-chain polysulfides are completely converted to Na_2_S. Therefore, it is necessary to design an advanced sulfur-loaded electrode to solve the shuttle problem of NaPSs.

So far, since the substantive problems of S cathodes have not been completely solved, the key research direction in the field of RT Na-S batteries is still to stabilize S cathodes through material structure design. At room temperature, the initial solid-state S of the battery cathode need to overcome a huge reaction activation energy to convert it into the final chemical product solid-state Na_2_S_2_/Na_2_S (Yu and Manthiram, 2020). This step is difficult in terms of redox kinetics, which directly leads to low utilization of active substances, less capacity output and low Coulombic efficiency. At the same time, ether-based and ester-based electrolytes, binders, sulfur loading, etc. will also affect the energy storage capacity of cathode materials (Li, et al., 2019). The following is an analysis of the problems existing in the S cathode structure from two aspects. 1) The cathode structure collapses: During the charging and discharging process of the battery, the sulfur electrode undergoes a “solid-liquid-solid” phase transition process. Due to the different densities of the intermediate product NaPSs, the sulfur-loaded material needs to withstand the influence of a relatively drastic volume change. The inevitable result of volume expansion and contraction is the collapse of the cathode material and the loss of active substances. The volume change as high as 171% when the active material is fully reacted and dissolved is the root cause of the rapid collapse of the electrode structure, which makes the battery unable to obtain longer cycle life. For example, the pores of some carbon materials are initially filled with full active substances, and after the volume expansion and structural shrinkage changes, only less than 60% of the content is seriously left. 2) Active substances are dissolved and released: Molecular dissolution of the intermediate Na_2_S_n_ generated during charging and discharging of RT Na-S batteries, such as long-chain Na_2_S_4_, Na_2_S_6_ and Na_2_S_8_, escape into the electrolyte during charging and discharging (compared to Li-S batteries, one more serious problem of Na-S batteries). In addition, short-chain polysulfides can be irreversibly deposited on the electrodes, resulting in rapid capacity fading of Na-S batteries and reduced Coulombic efficiency. Long-chain polysulfides are readily soluble in organic electrolytes and diffuse in organic electrolytes, resulting in the loss of active sulfur (Lei, et al., 2022a), a phenomenon called the “shuttle effect”. At the same time, the insulating nature of sulfur and the formation of discharge product Na_2_S_2_/Na_2_S also lead to insufficient reaction kinetics of sodium ion diffusion and high polarization (Yan, et al., 2021). The utilization rate of the active substances sulfur is low, resulting in a decrease in the Coulombic efficiency. In addition, the conversion of sulfur species during the redox reaction in RT Na-S batteries are more difficult than that in lithium-sulfur batteries in terms of reaction kinetics due to the larger radius of sodium ions (Mou, et al., 2021). To make matters worse, the slower conversion reaction rate would bring the accumulation of soluble NaPSs, thus deepening the shuttle effect.

**FIGURE 1:**
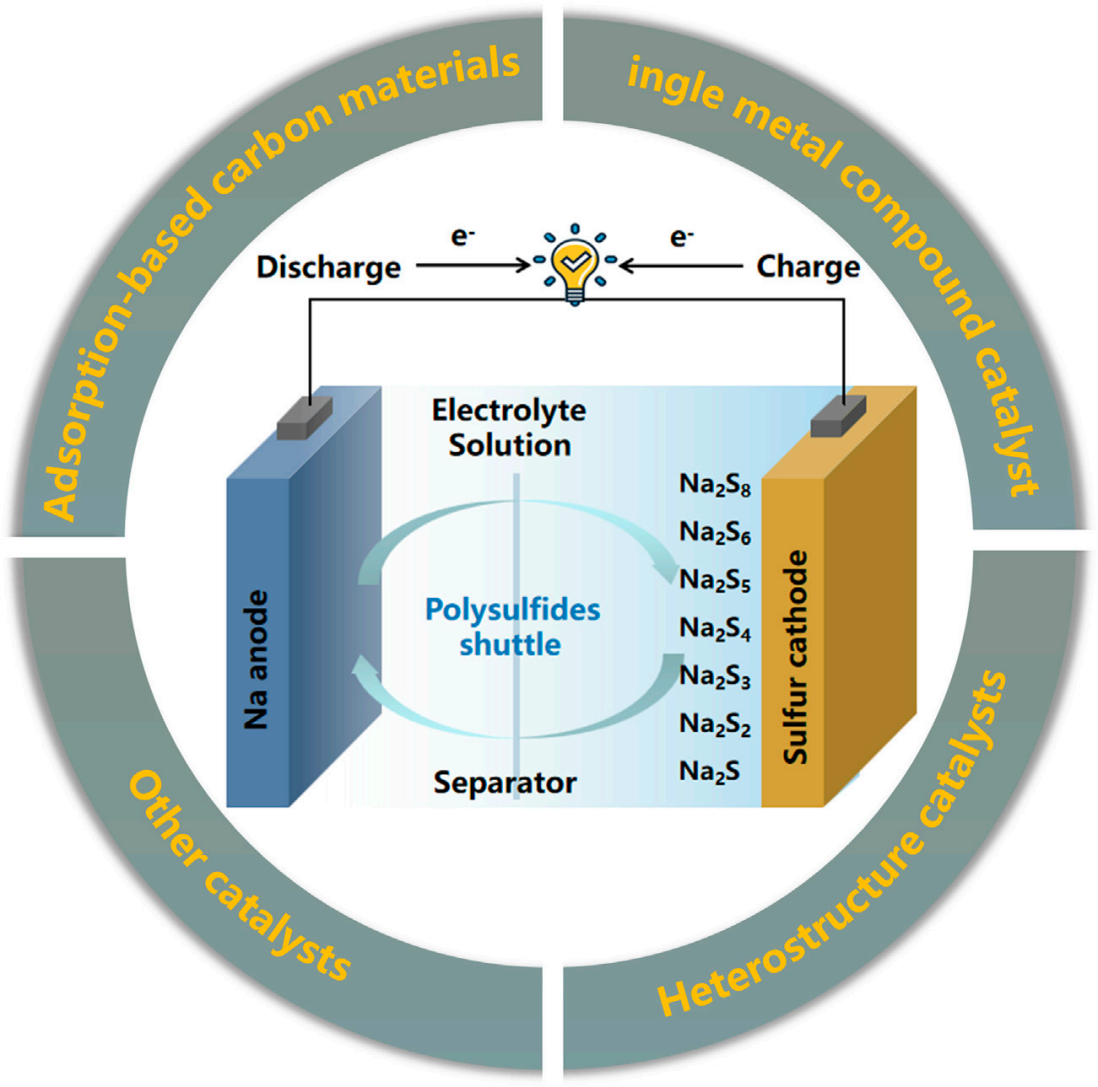
Schematic diagram of the construction strategy of carbon-based cathode materials for room temperature sodium-sulfur batteries (Wang, et al., 2021a).

Overall, the reason why it is difficult to achieve high utilization of S cathode is not only related to the dissolution of intermediate products due to contact with the electrolyte, but also related to its own structural characteristics and reaction mechanism. Therefore, according to the current relatively novel research reports, to improve the cycle stability of cathode materials, the cathode composite materials for RT Na-S batteries can be constructed based on both adsorption and electrocatalysis (Yang, et al., 2021b; Yang, et al., 2021c). This paper summarizes the main factors that lead to the instability of cathode materials for RT Na-S batteries, and introduces long-term effective optimization strategies for cathode materials. According to the optimization strategy for RT Na-S battery cathode materials based on carbon-based materials introduced in the review (Figure 1), some points for further research are proposed, and the follow-up research on battery stability improvement strategies is prospected. This paper provides a very intuitive way for beginners to understand the structural design principles of cathode materials for RT Na-S batteries.

## 2 Construction of Carbon-Based Composite Cathode Materials

It is well known that the shuttle behavior of sodium polysulphides (NaPSs) is a transition from the high concentration side to the low concentration side. Therefore, confining NaPSs in the cathode region by blocking or controlling the diffusion pathway of soluble NaPSs is the most direct and effective way to eliminate the shuttle effect. Therefore, in order to suppress the shuttle effect caused by the dissolution of NaPSs into the organic electrolyte, promote the redox reaction of sulfur, and at the same time improve the overall electrochemical performance of RT Na-S cells, carbon-based metal compounds with carbon substrate as the main component were used to construct the composite cathode material for RT Na-S battery has a wide research space and profound research value (Xiao, et al., 2021b). The carbon matrix as the main nanostructured cathode material has a special geometric space and a large specific surface area to accommodate the S active substances, and the excellent electrical conductivity is conducive to the transition of electrons and ions. As NaPSs storage spaces, these hosts can physically adsorb NaPSs species through the most fundamental van der Waals forces and confine them in well-designed porous spaces. In addition, some materials with porous carbon shell structures can also act as barriers to hinder the dissolution and escape of NaPSs. At the same time, the addition of metal compounds is a more important link (Huang, et al., 2021b). In addition to the carbon matrix composites that play an adsorption role, the composite electrode materials after the introduction of metal compounds can accelerate the NaPSs conversion process, which is a key way to avoid the accumulation of soluble NaPSs and alleviate the severe shuttle effect (Dai, et al., 2021). In detail, when the metal compound catalyst is added to the sulfur cathode, the soluble NaPSs will be adsorbed by the electrocatalyst, and the charge in the electrocatalyst will accelerate the redox reaction of NaPSs, thereby reducing the time that NaPSs is in a dissolved state. In the follow-up, this paper will analyze and discuss the carbon-based metal compound composite cathode materials based on the specific research results in recent years (Wang, et al., 2021b).

### 2.1 Construction of RT Na-S Battery Cathode Materials Based on Adsorption

**FIGURE 2:**
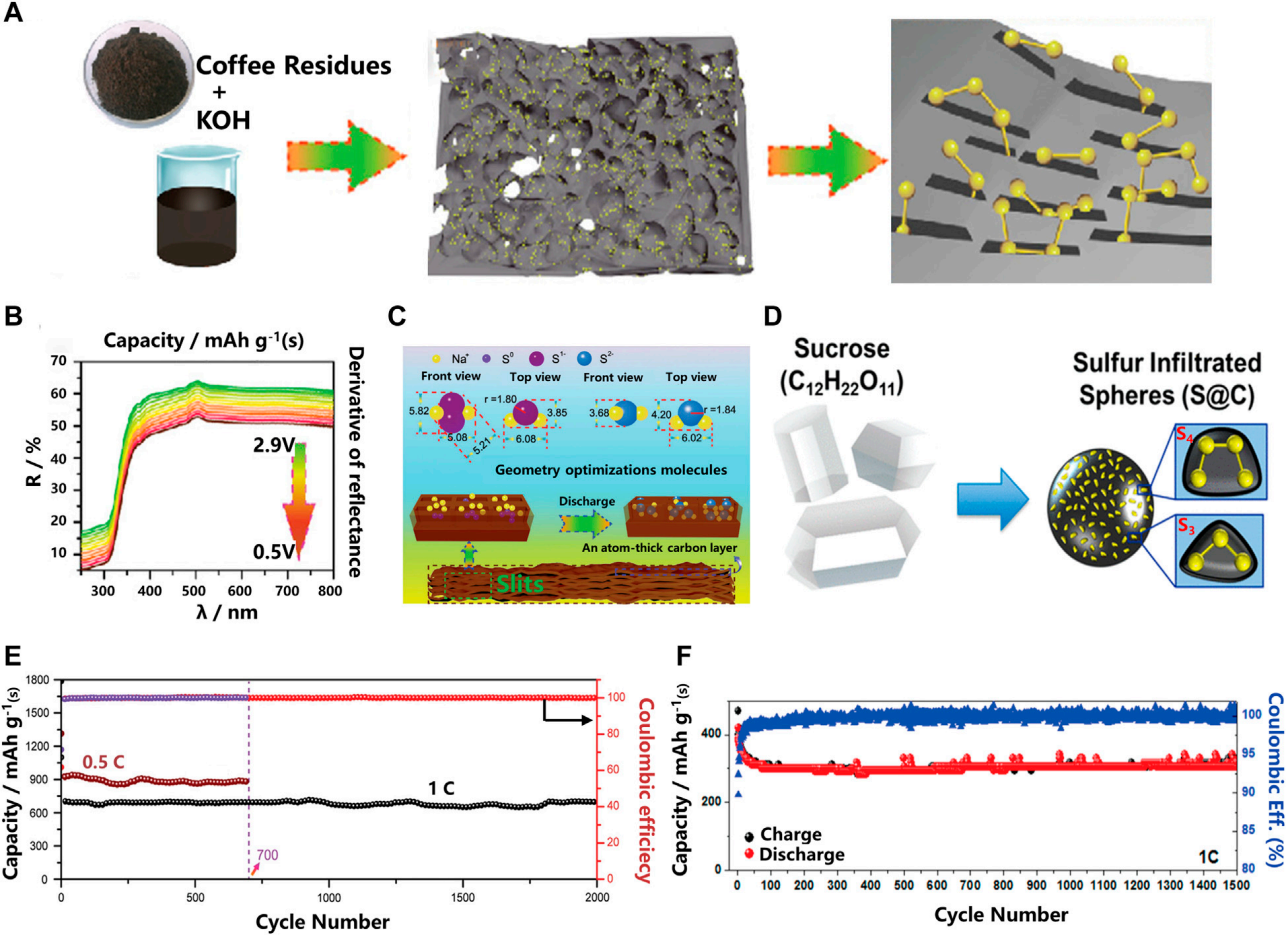
**(A)** Schematic illustration of the synthesis procedure for the microporous carbon and sulfur composite with small sulfur molecules confined in the carbon micropores; **(B)** *In situ* UV-vis spectra; **(C)** Schematic illustration of the reaction mechanism of the ACC-40S cathode for RT Na–S batteries; **(D)** Schematic representation of the material processing steps of using sucrose (sugar) to produce microporous sodium sulfur battery cathodes; **(E)** Cycle performances of the ACC-40S electrode at 0.5 and 1 C for 700 and 2000 cycles, respectively; **(F)** Prolonged cycling performance of 1,500 cycles at 1C of S@C (Carter, et al., 2017; Guo, et al., 2020).

In RT Na-S batteries, carbon is the most commonly used sulfur host material, which can adsorb higher-order NaPSs within the cathode material to prevent their dissolution and improve the slow kinetics of reversible conversion of short-chain polysulfides. To improve the overall electrochemical performance of RT Na-S batteries, different types of carbon materials such as hollow carbon microspheres and nanocarbon matrices or their composites have been prepared. For example, confinement of small sulfur molecules with a microporous carbon host precludes the dissolution of long-chain NaPSs by forming only short-chain NaPSs but still achieves high capacity retention and cycling stability. As shown in Figures 2A–C, Qiubo Guo et al. reported an efficient small sulfur molecular carrier using coffee grounds-derived active ultra-micropores with a micropore size of about 0.5 nm and a specific surface area of up to 1,100 m^2^ g^−1^ (Guo, et al., 2020) (Figure 2E). The experimental results show that the carbon-sulfur composite cathode is prepared by dispersing commercially available sulfur into ultra-microporous materials by the traditional melt-diffusion method. It is worth noting that the active substances must be located inside the void rather than outside. The full battery still has a capacity retention rate of 1,492 mAhg^−1^ after 2000 cycles at a rate of 0.1C, with almost no capacity decay. Simultaneous density functional theory (DFT) calculations show that small sulfur molecules (S_2–4_) exist in slit ultra-micropores with a diameter of 0.5 nm. Electrochemical tests as well as *ex-situ* and *in-situ* characterizations, such as *in-situ* UV-*vis* spectroscopy, further confirmed the one-step reaction mechanism of the only reduction product Na_2_S, indicating that this carbon-based material effectively adsorbs long-chain polysulfides. However, when small sulfur molecules are encapsulated into the microporous carbon matrix, only a limited amount of active substances sulfur (less than 40%) can be loaded, resulting in a low energy density of the full cell.

Among various carbon matrix material morphologies, microporous carbon materials have unique reaction mechanisms compared with ultra-microporous materials, and thus exhibit the highest practical potential. Using sucrose as the carbon source, Rachel Carter et al. (Carter, et al., 2017) prepared ordered microporous carbon spheres with a pore size of 0.5 nm as the sulfur carrier for the cathode of RT Na-S batteries (in Figure 2D). The as-prepared cathode exhibits good electrochemical performance, indicating the feasibility of micropore confinement. At a rate of 1C, the reversible capacity of the cathode remains 300 mAhg^−1^ after 1,500 cycles, and the Coulombic efficiency is as high as 98% (Figure 2F). The above analysis shows that the special pore structure of the carbon-based composite cathode material can inhibit the dissolution of soluble NaPSs through the mechanism of adsorption (Fan, et al., 2016; Jeon, et al., 2020; Murugan, et al., 2021), thereby providing a longer cycle life of the battery.

### 2.2 Construction of Cathode Materials for RT Na-S Batteries Based on Electrocatalysis

In addition to the traditional adsorption-based carbon material composite cathodes, the introduction of electrocatalysts within the cathode plays a key role in promoting the NaPSs redox reaction, especially the conversion from long-chain polysulfides to the final product Na_2_S (Wang, et al., 2021c). In recent years, researchers have designed a variety of electrocatalysts to improve the utilization and rate capability of RT Na-S batteries.

#### 2.2.1 Single-Component Metal Compounds

**FIGURE 3:**
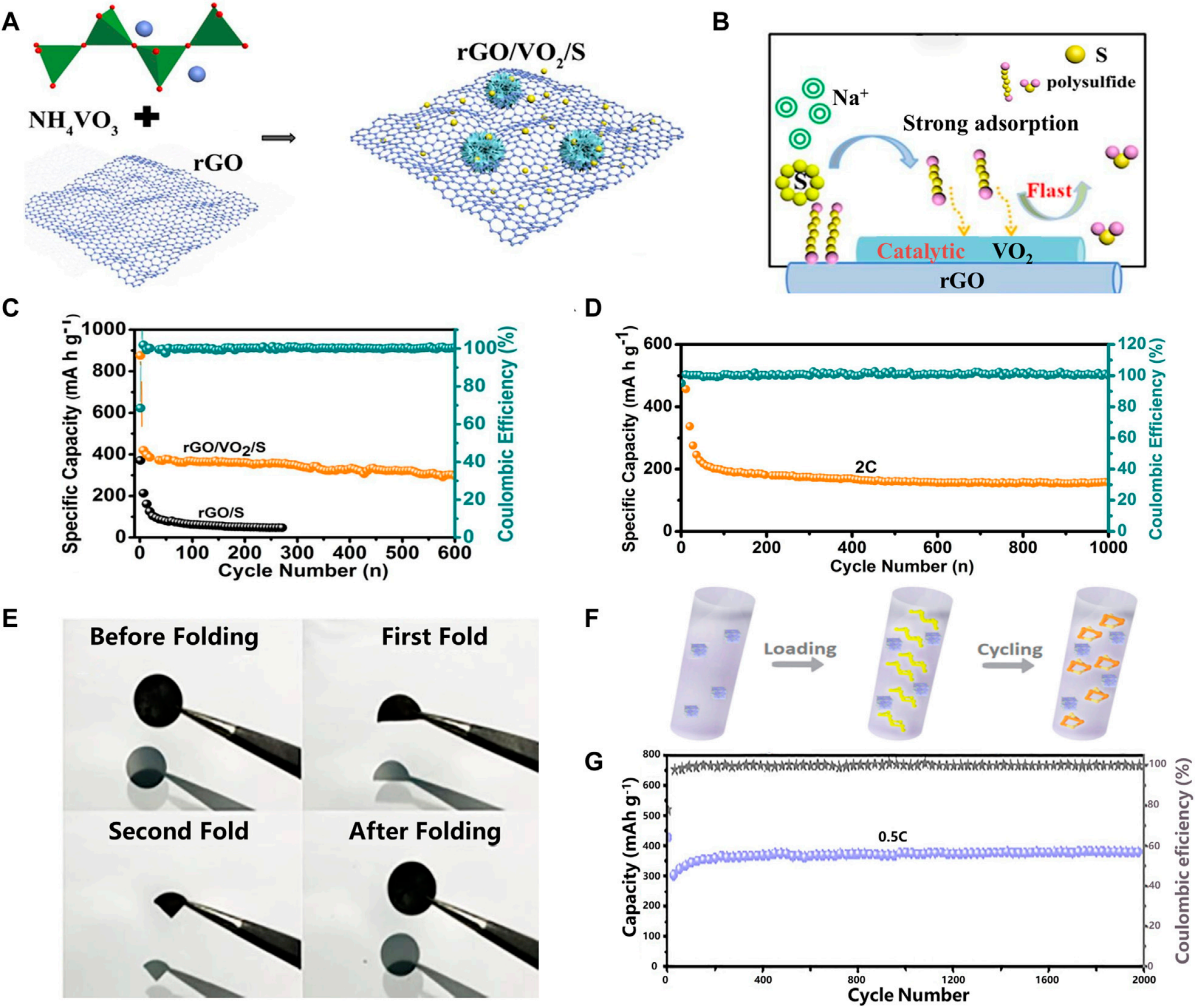
Schematic of the **(A)**preparation and **(B)** advantages of the rGO/VO_2_/S composite; **(C)** Cycling performance and Coulombic efficiency at 0.2C; **(D)** Cycling stability at 2C for 1,000 cycles; **(E)** Digital photos of the VC-CNFs as a freestanding electrode; **(F)** VC-CNFs@S cathode; **(G)** Prolonged cycling performance at 0.5C (activated at 0.1C) (Du, et al., 2020; Tang, et al., 2020).

Some metal compounds can be used as catalysts on the cathode side of RT Na-S (Qi, et al., 2021). In addition to electrocatalysis, metal oxides can also adsorb NaPSs due to the strong electronegativity of O atoms. For example, a three-dimensional porous structure composite was prepared by a hydrothermal method (in Figure 3A) by Wenyan Du et al. A three dimensional (3D) hierarchical cathode substrate with VO_2_ nanoflowers as catalyst *in situ* grown on reduced graphene oxide (rGO) for sulfur cathodes are designed and prepared (Du, et al., 2020). Compared with the rGO/S composites (without VO_2_ nanoflowers by a hydrothermal method), the rGO/VO_2_/S composites exhibited smaller charge transfer resistance and a negligible shuttle effect (Figure 3B). It can be also seen from the high repeatability of the CV curves and the charge transfer resistance reaction kinetics that rGO/VO_2_/S cathode exhibits good cycling stability and effectively enhances the reaction kinetics process and promotes the conversion of polysulfides. In Figures 3C,D, when the current density is 0.2 C, the initial reversible capacity of the rGO/VO_2_/S composite is 876.4 mAh g^−1^, and the capacity of 400 mAh g^−1^ is still maintained after 200 cycles. The long-term cycling performance of cathode also retained the discharge capacity of 156 mAh g^−1^ after 1,000 cycles with a high Coulombic efficiency of over 99%. Other metal compounds, such as VC, also exhibit excellent catalytic performance and electron transport acceleration effect, inhibiting the shuttle of NaPSs. Wenwen Tang et al. (Tang, et al., 2020) encapsulated VC nanoparticles in uniform carbon fiber nanotubes (CNFs) by electrospinning, and then prepared VC-CNFs@S self-supporting electrodes by traditional melt diffusion method (Figures 3E–G). Two distinct cathodic peaks in the cyclic voltammogram of the VC-CNFs electrode indicate the reduction of sulfur to polysulfides and the reduction of polysulfides to Na_2_S, respectively. The charge transfer resistance of the VC-CNFs electrode is much smaller than CNFs electrode, demonstrating faster charge transfer behavior at the VC-CNFs/polysulfides interface, which can significantly enhance the redox kinetics of polysulfides. During the charge-discharge process, NaPSs were captured and adsorbed by VC nanoparticles and catalyzed and accelerated their redox reactions, thus avoiding the diffusion of soluble NaPSs. The battery performance is shown as 379 mAh g^−1^ capacity retention after 2000 cycles at a rate of 0.5 C, with a capacity retention rate of 96.2%.

#### 2.2.2 Two-Component Metal Compounds

**FIGURE 4:**
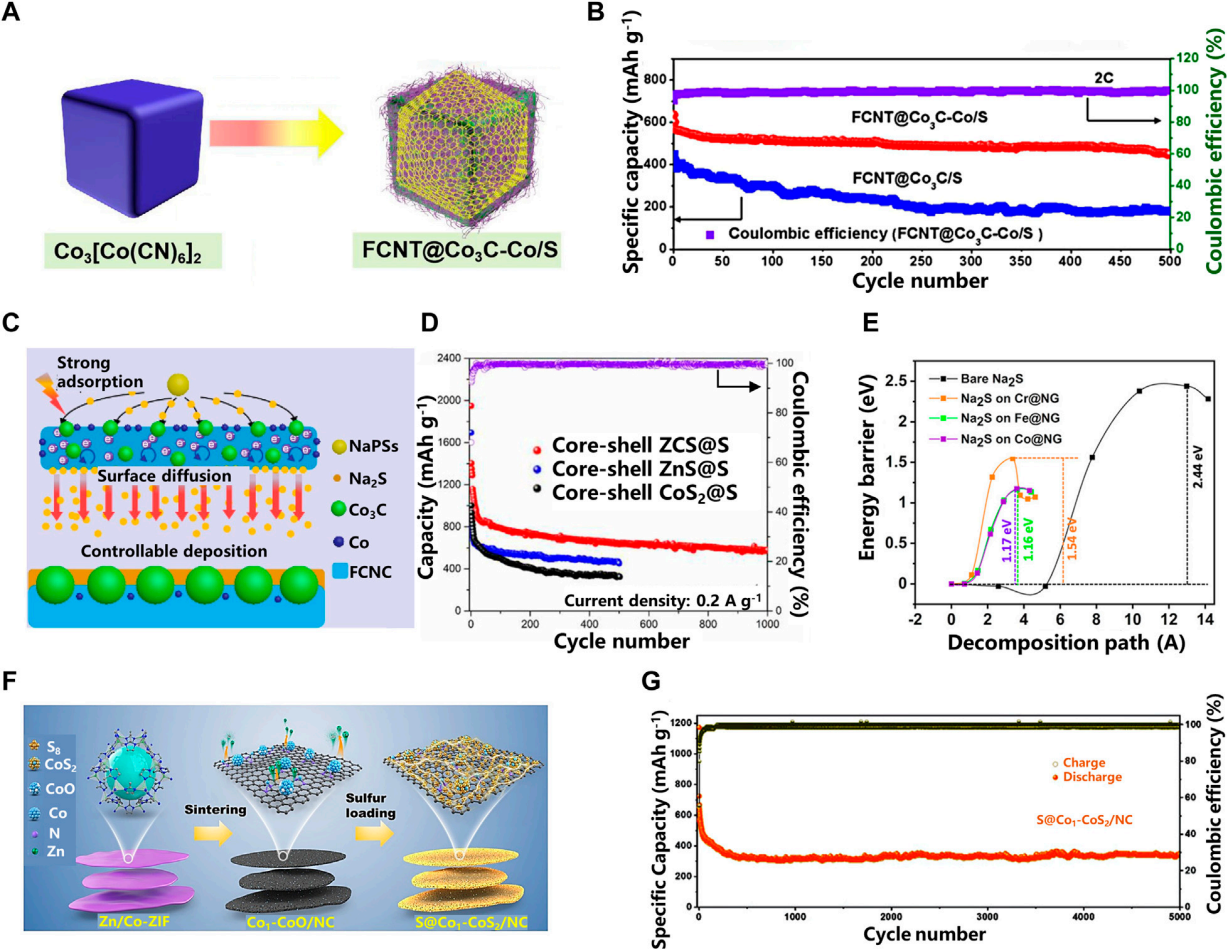
**(A)** The Schematic of the Preparation of FCNT@Co_3_C-Co/S; **(B)** The long cycling performances for FCNT@Co_3_C/S and FCNT@Co_3_C-Co/S at 2C; **(C)** The schematic diagram illustration of the Na_2_S deposition and growth process on FCNT@Co_3_C-Co/S; **(D)** Cycling performances at 0.2 A g^−1^ of different cathode materials; **(E)** Decomposition barrier of bare Na_2_S and Na_2_S on TM@NG substrates; **(F)** Illustration of synthetic strategy to produce S@Co_1_−CoS_2_/NC; **(G)** Long-term cycling stability of S@Co_1_−CoS_2_/NC at the current density of 5 A g^−1^ (Liu, et al., 2020; Qin, et al., 2020; Jayan and Islam, 2021).

In addition, in order to integrate the common advantages of multiple materials, a hybrid design of carbon-based cathode materials for RT Na-S batteries can be performed, and the heterostructure has strong adsorption capacity and good catalytic conversion performance (Wu, et al., 2021). For example, by adjusting the doping ratio of Co atoms, enhanced synergistic and catalytic effects can be achieved. Guohui Qin et al. (Qin, et al., 2020) synthesized Co_3_C-Co co-doped fluorinated carbon nanotubes (FCNT@Co_3_C-Co) (in Figure 4A). This shows that the FCNT@Co_3_C-Co/S electrode containing 5% Co exhibits an extremely high discharge capacity of 1,364 mAh g^−1^ at 0.1 C. After 500 cycles at a rate of 2 C, the FCNT@Co_3_C-Co/S electrode can still maintain a residual capacity of 79% (in Figure 4B). The results show that proper Co doping makes the SEI film between solid electrolytes more stable. With the increase or decrease of the controlled Co ratio, the Coulombic efficiency of FCNT@Co_3_C/S decreases rapidly, from 98% in the 20th cycle to 45% in the 100th cycle, indicating that Co plays an important role in achieving higher Coulombic efficiency. In Figure 4C, the synergistic effect of Co_3_C-Co lowers the nucleation energy barrier of NaPSs and the impedance of redox reaction, and accelerates the conversion of soluble long-chain polysulfides to insoluble short-chain polysulfides.

Similarly, Hanwen Liu et al. (Liu, et al., 2020) compared the electrochemical performance of Co-doped, Zn-doped and Zn/Co co-doped carbon-based sulfur electrodes (ZCS). They found that the combination of Zn and Co atoms can lower the energy barrier for redox reactions and work synergistically to suppress the shuttle effect. Through the charge transfer resistance histogram, it can be intuitively found that the charge transfer resistance of ZCS@S electrode is smaller than that of the other two electrodes. The potential barrier (2.6 V) of ZCS@S electrode is lower than that of ZnS@ S electrode and CoS_2_@S electrode, indicating the existence of thiophilic sites of Zn-Co atoms and can lower the potential barrier for Na_2_S to long-chain polysulfides conversion. The combination of NaPSs with ZnS and CoS_2_ has a good catalytic effect and exhibits good electrochemical performance. After 1,000 cycles at a current density of 0.2 A g^−1^, the ZCS@S electrode still maintains a reversible capacity of 570 mAh g^−1^ (Figure 4D).

#### 2.2.3 Others

Although metal compound composite electrocatalysts have good electrocatalytic performance, sometimes in order to pursue materials with higher catalytic activity, some single metal catalysts are also applied to composite cathode materials for RT Na-S batteries (Qi, et al., 2020; Ye, et al., 2021; Zhang, et al., 2021). Rahul Jayan et al.(Jayan and Islam, 2021) elucidate the adsorption and binding energies of NaPSs in a novel single-atom catalyst and battery system embedded on nitrogen-doped graphene by density functional theory (DFT) calculations. Compared with the nitrogen-doped graphene material without transition metal single atoms, it is found that the single-atom catalyst has strong chemical interaction with NaPSs (in Figure 4E).

On the basis of previous work, Yaojie Lei et al. (Lei, et al., 2022b) recently used cobalt sulfide as an electron reservoir to enhance the activity of the sulfur cathode, while combining with cobalt single atoms as double-terminal binding sites to achieve a stable sulfur conversion process. Two-dimensional N-doped carbon matrix (NC) decorated with polar cobalt sulfide (CoS_2_) and single-atom Co (Co_1_) can serve as a multifunctional sulfur host (S@Co_1_- CoS_2_/NC) (in Figure 4F). The rationally constructed CoS_2_ electron reservoir is capable of directly reducing S to short-chain sodium polysulfide (Na_2_S_4_) via a streamlined redox pathway of electron transfer. At the same time, cobalt single atoms and electron pools work synergistically to further strengthen the streamlined redox pathway, immobilize the long-chain products formed *in situ* and catalyze their transformation, thereby achieving high sulfur utilization and sustainable cycling stability. The developed sulfur cathode exhibits an excellent rate capability of 443 mAh g^−1^ at 5 A g^−1^ and still has a high cycle capacity retention rate of 80% after 5,000 cycles at 5 A g^−1^ (in Figure 4G).

## 3 Conclusion and Perspectives

In summary, this paper reviews the research progress in the construction of carbon-based metal compound composite cathode materials for RT Na-S batteries. The design of support structures in two general directions is summarized by the structural analysis of sulfur cathodes, including studies based on adsorption and electrocatalytic conversion of NaPSs by metallides. The headaches inherent in RT Na-S battery cathodes are largely alleviated. Therefore, summarizing the design strategy of long-term performance and stability of cathode materials for RT Na-S batteries is mainly to solve three main problems, namely, high-strength stable structure, inhibition of NaPSs dissolution, and acceleration of redox kinetics.

However, there are still some unavoidable problems in the above-mentioned cathode material optimization scheme, and further research and development are needed to put RT Na-S batteries into practical applications on a large scale. Although this paper summarizes the research progress of adsorption catalysis for RT Na-S batteries, the existing metal-based catalysts still have defects. For example, the poor electrical conductivity of metal compound electrocatalysts has a negative impact on its catalytic ability, and how to control the catalytic activity so that the catalyst can play a proper role is still the crux of the matter. Therefore, it is necessary to strengthen corresponding theoretical calculations and *in situ* characterization methods to assist in better design and regulation of catalytic performance. In terms of battery performance, the high amount of active material surface loading determines the available capacity of the battery, but the problem of serious capacity loss will inevitably occur. The ultimate goal is to maintain high capacity and efficient capacity together.
